# Paradoxical Reaction to Antitubercular Treatment in a Case of Tuberculous Meningitis

**DOI:** 10.7759/cureus.44151

**Published:** 2023-08-26

**Authors:** Solangaratchige Don Harshana Sameera Perera

**Affiliations:** 1 Respiratory Medicine, Central Chest Clinic, Colombo, LKA

**Keywords:** anti-tubercular treatment, meningitis, cervical lymphadenopathy, central nervous system tuberculosis, paradoxical reaction

## Abstract

Tuberculous meningitis (TBM) is a challenging disease to treat, as *Mycobacterium tuberculosis *infects the meninges, which are the outer membranes of the brain and the spinal cord. The majority of patients diagnosed with TBM acquire various other central nervous system complications, and as a result, treating the disease becomes a challenging task. A paradoxical reaction to the treatment may occur in the course of managing TBM. This case study describes a 20-year-old Southeast Asian female who was diagnosed and treated for TBM and subsequently developed a resurgence of the disease due to a paradoxical reaction.

## Introduction

Tuberculous meningitis (TBM) is an infection in the central nervous system that is challenging to treat and is associated with significant morbidity and mortality. Sri Lanka has a high tuberculosis (TB) burden; however, the national control program has successfully controlled the incidence in the last decade. The incidence rate of TB infection in Sri Lanka was 29.1 per 100,000 people in 2021 [1], while TBM accounts for 4.9% of the total incidence. The global TB incidence in 2021 was reported at 10.6 million [2]

“Paradoxical Reaction” (PR) during antituberculous treatment (ATT) occurs due to a complex interplay between the host’s immune response and the direct effect of mycobacterial antigens [3]. This phenomenon might worsen the existing disease or lead to the appearance of new lesions. The timing of this reaction is unpredictable, and it has been reported more frequently following the commencement of the treatment [4].

This study describes a patient who was on treatment for TBM and later suffered worsening and new symptoms due to a paradoxical reaction. Therefore, the study depicts the importance of paradoxical reactions as a differential diagnosis in patients not responding to ATT.

## Case presentation

A 20-year-old Southeast Asian unmarried female, with a contact history of pulmonary tuberculosis, presented with photophobia, fever, and headaches for two weeks. The patient’s cerebrospinal fluid (CSF) analysis revealed 165 polymorphs, 43 lymphocytes, protein 230 mg/dl, glucose 1.7 mmol/L (RBS 5.2 mmol/L), with cytology showing lymphocytic predominant mixed filtrate. However, the CSF TB polymerase chain reaction (PCR) was negative. With her erythrocyte sedimentation rate (ESR) being persistently high and Mantoux reading being between 10 mm and 15 mm on multiple occasions, she was started on ATT with a provisional diagnosis of TBM. The diagnosis was further substantiated by an MRI brain study, which depicted multiple ring-enhancing lesions suggestive of TB granuloma.

The patient successfully completed the two-month initiation phase of ATT. However, in the third month of the continuation phase of ATT therapy, she presented with progressive enlargement of a single right anterior cervical lymph node along with a weight loss of 2 kg and loss of appetite for one week. The aforementioned lymph node gradually increased in size and measured 1.0 cm to 2.0 cm in size at the time of presentation. The nodule was firm and tender, and the overlying skin was mildly erythematous. She did not complain of any other symptoms, and her cardiovascular, respiratory, gastrointestinal, musculoskeletal, and genitourinary system examinations were normal. In order to exclude other causes for her headache and photophobia apart from meningeal irritation, she was directed to ear-nose-throat (ENT) and ophthalmology examinations but was found to be normal. 

Various blood and radiological investigations were then performed. Her repeated full blood counts had a high lymphocytic count, while the ESR and C-reactive protein (CRP) levels were persistently high. Moreover, her peripheral blood smear showed normocytic and normochromic red cells with marked rouleaux formation. The white cells were normal in appearance, with neutrophils showing toxic changes. Platelets were abundant, suggesting infection or inflammation, but there was no evidence of lymphoma. Serum electrolytes, renal functions, and liver functions were normal, and urine, blood, and sputum cultures were also normal. Her Mantoux test was positive on each occasion, despite it being non-specific in diagnosing TB; the sputum investigations conducted which included culture, Gene Xpert *Mycobacterium tuberculosis */Rifampicin (MTB/RIF) assay, and direct microscopy for acid-fast bacilli (AFB), were negative. Moreover, she was screened for diseases causing immunosuppression including the HIV status, which also revealed negative results.

**Figure 1:**
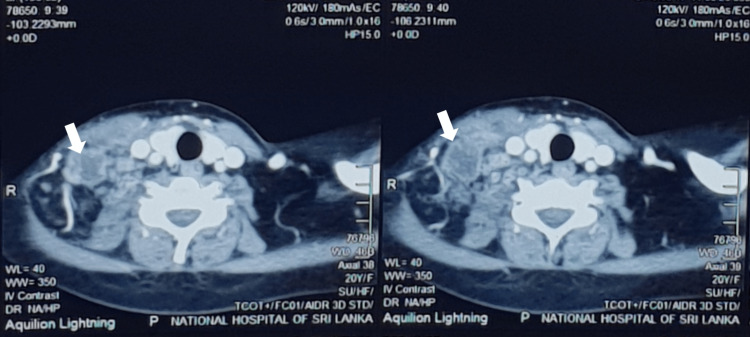
CECT chest and neck CECT: contrast-enhanced computed tomography Arrows indicate right level 4 anterior cervical lymphadenopathy

A chest X-ray showed no abnormalities. However, the ultrasound of the neck revealed an irregular, heterogeneously hypoechoic lesion with some echogenic material in the right level 4 cervical region measuring 3.5 cm to 3.0 cm in size, suggesting an infective appearance. Additionally, the neck and chest contrast-enhanced computed tomography (CECT) (Figure 1) showed an enlarged cervical lymph node at level 4 on the right side, measuring 3.9 cm to 3.7 cm. with necrotic and cystic changes within it. The left mediastinal level 1 lymph node was noted to have a central necrotic area, measuring 1.7 cm to 1.1 cm in size.

A fine needle aspiration cytology (FNAC) of the right cervical lymph node revealed granular material mixed with scattered neutrophils, on a lymphoid background with no Langerhans-type giant cells or granulomata [5]. AFB culture and bacterial culture, along with the Gene Xpert MTB/RIF of the fine needle aspiration, were not significant, which prompted a biopsy of the lymph node. The biopsy histology revealed granulomatous inflammation with focal suppuration, and the rapid liquid culture isolated MTB. Additionally, endobronchial ultrasonography (EBUS) showed another enlarged mediastinal lymph node mass extending from 2R to 4R, and samples were taken from it. The patient's histology and cytology showed the characteristic feature of MTB infection, and the Gene Xpert MTB/RIF assay also detected MTB.

The patient presented with the above symptoms while receiving treatment for tuberculous meningitis. She had successfully completed two months of the initiation phase of ATT [6] with isoniazid (H), rifampicin (R), pyrazinamide (Z), ethambutol (E), and streptomycin (S) for two months, and three months of the continuation phase (total of 10 months) with H and R. As her cervical lymph node and mediastinal lymph node isolated MTB, the patient was re-started on ATT therapy under a retreatment regime [7]. The novel regime was introduced with H, R, Z, E for two months, H, R, E for three months, and H, R for five months. Simultaneously she was started on oral Dexametazone for the initial two months on a tapering scheme.

## Discussion

Paradoxical reactions have been common in the treatment of MTB in the last decade. However, on most occasions, it is either unnoticed or considered a treatment failure or relapse. The patient in this case was previously diagnosed with TBM and successfully treated with ATT drugs. However, in the 3rd month of the continuation phase, she was noticed to have cervical and mediastinal lymphadenitis, which was laboratory-diagnosed to be secondary to MTB.

A novel infection of tuberculosis on two different sites in a patient already on ATT therapy, combined with the recent infection persisting irrespective of the ongoing treatment with the exclusion of other possible differentials, led to the diagnosis of a paradoxical reaction while on treatment for TBM. This diagnosis was further confirmed by the patient responding to a short course of oral steroids [8] and a retreatment ATT regime, along with subsequent investigations including CSF analysis, PCR, cultures, cytology, and histology showing normal results.

Drug-resistant TB, lymphoma, mycobacterium other than tuberculosis (MOTT) infection, and treatment failure were the main differentials regarding this patient. This case report was mainly written targeting clinicians to always consider paradoxical reactions as a differential while treating challenging tuberculous infections.

## Conclusions

Clinicians practicing in the third-world countries frequently receive patients with different forms of tuberculous infections. Poor living conditions, and lack of compliance to treatment, have been contributing to the above. Therefore, it is vital to closely follow up the patients throughout the course of their treatment. The phenomenon of paradoxical reaction is defined as worsening of clinical or radiological findings following the initiation of appropriate ATT with no evidence of disease relapse or a second diagnosis. The timing of the reaction varies with the patient and the treatment regime. However, treatment for TB lymphadenitis and cerebral disease have been reported as having the most complicated paradoxical reactions. Therefore, physicians should be vigilant for paradoxical reactions in the treatment of tuberculous disease.
